# Reinvestigation of trilithium divanadium(III) tris­(orthophosphate), Li_3_V_2_(PO_4_)_3_, based on single-crystal X-ray data

**DOI:** 10.1107/S1600536813001499

**Published:** 2013-01-19

**Authors:** Yongho Kee, Hoseop Yun

**Affiliations:** a Division of Energy Systems Research and Department of Chemistry, Ajou University, Suwon 443-749, Republic of Korea

## Abstract

The structure of Li_3_V_2_(PO_4_)_3_ has been reinvestigated from single-crystal X-ray data. Although the results of the previous studies (all based on powder diffraction data) are comparable with our redetermination, all atoms were refined with anisotropic displacement parameters in the current study, and the resulting bond lengths are more accurate than those determined from powder diffraction data. The title compound adopts the Li_3_Fe_2_(PO_4_)_3_ structure type. The structure is composed of VO_6_ octa­hedra and PO_4_ tetra­hedra by sharing O atoms to form the three-dimensional anionic framework _∞_^3^[V_2_(PO_4_)_3_]^3−^. The positions of the Li^+^ ions in the empty channels can vary depending on the synthetic conditions. Bond-valence-sum calculations showed structures that are similar to the results of the present study seem to be more stable compared with others. The classical charge balance of the title compound can be represented as [Li^+^]_3_[V^3+^]_2_[P^5+^]_3_[O^2−^]_12_.

## Related literature

For the isotypic Li_3_Fe_2_(PO_4_)_3_ structure, see: Patoux *et al.* (2003 ▶). Structural studies of Li_3_V_2_(PO_4_)_3_ based on powder diffraction data have been reported previously by Yin *et al.* (2003 ▶); Patoux *et al.* (2003 ▶); Kuo *et al.* (2008 ▶); Yang *et al.* (2010 ▶); Fu *et al.* (2010 ▶). For ionic radii, see: Shannon (1976 ▶). For bond-valence calculations, see: Adams (2001 ▶). For the Inorganic Crystal Structure Database, see: ICSD (2012 ▶).

## Experimental

### 

#### Crystal data

Li_3_V_2_(PO_4_)_3_
*M* *_r_* = 407.61
Monoclinic, 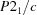

*a* = 8.6201 (4) Å
*b* = 8.6013 (4) Å
*c* = 14.7465 (7) Å
β = 125.204 (3)°
*V* = 893.39 (7) Å^3^
*Z* = 4
Mo *K*α radiation
μ = 2.70 mm^−1^
*T* = 290 K
0.08 × 0.04 × 0.04 mm

#### Data collection

Rigaku R-AXIS RAPID diffractometer
Absorption correction: multi-scan (*ABSCOR*; Higashi, 1995 ▶) *T*_min_ = 0.741, *T*_max_ = 1.000
8328 measured reflections
2031 independent reflections
1772 reflections with *I* > 2σ(*I*)
*R*_int_ = 0.031

#### Refinement

*R*[*F* ^2^ > 2σ(*F* ^2^)] = 0.024
*wR*(*F* ^2^) = 0.062
*S* = 1.08
2031 reflections
181 parameters
Δρ_max_ = 0.58 e Å^−3^
Δρ_min_ = −0.49 e Å^−3^

### 

Data collection: *RAPID-AUTO* (Rigaku, 2006 ▶); cell refinement: *RAPID-AUTO*; data reduction: *RAPID-AUTO*; program(s) used to solve structure: *SHELXS97* (Sheldrick, 2008 ▶); program(s) used to refine structure: *SHELXL97* (Sheldrick, 2008 ▶); molecular graphics: *DIAMOND* (Brandenburg, 1999 ▶); software used to prepare material for publication: *WinGX* (Farrugia, 2012 ▶).

## Supplementary Material

Crystal structure: contains datablock(s) global, I. DOI: 10.1107/S1600536813001499/wm2716sup1.cif

Structure factors: contains datablock(s) I. DOI: 10.1107/S1600536813001499/wm2716Isup2.hkl

Additional supplementary materials:  crystallographic information; 3D view; checkCIF report

## Figures and Tables

**Table 1:** Selected bond lengths (Å)

Li1—O4	1.930 (6)
Li1—O5	1.940 (6)
Li1—O3^i^	2.094 (6)
Li1—O10^ii^	2.215 (6)
Li2—O3	1.968 (6)
Li2—O1	1.974 (6)
Li2—O7	2.004 (6)
Li2—O9	2.153 (7)
Li3—O6	1.944 (5)
Li3—O10	1.946 (5)
Li3—O11	1.994 (5)
Li3—O9	2.014 (6)
V1—O2	1.9040 (19)
V1—O8	1.954 (2)
V1—O1^i^	2.0163 (19)
V1—O7	2.0168 (18)
V1—O5	2.0450 (18)
V1—O3^i^	2.1172 (18)
V2—O12^iii^	1.9099 (19)
V2—O6	1.9810 (18)
V2—O4	2.0012 (18)
V2—O11	2.0316 (18)
V2—O10^ii^	2.0343 (19)
V2—O9^ii^	2.0618 (18)
P1—O2^iv^	1.5199 (19)
P1—O4^v^	1.5297 (19)
P1—O1	1.5310 (19)
P1—O6	1.5400 (18)
P2—O8^vi^	1.492 (2)
P2—O9	1.5419 (19)
P2—O7	1.5458 (18)
P2—O10^ii^	1.5497 (19)
P3—O12	1.5101 (19)
P3—O11	1.5343 (19)
P3—O5^vii^	1.5454 (19)
P3—O3^ii^	1.5506 (18)

Symmetry codes: (i) 
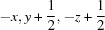
; (ii) 
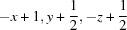
; (iii) 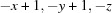; (iv) 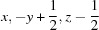; (v) 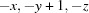; (vi) 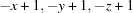; (vii) 
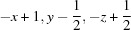
.

**Fig. 1.**
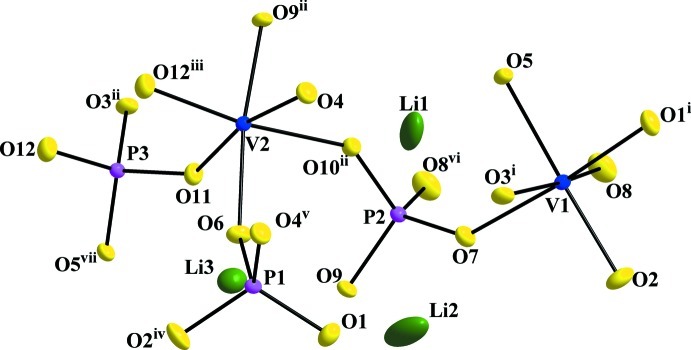
A view showing the local coordination environments of the V and P atoms with the atom labelling scheme. Displacement ellipsoids are drawn at the 50% probability level. Symmetry codes are as given in Table 1.

**Fig. 2.**
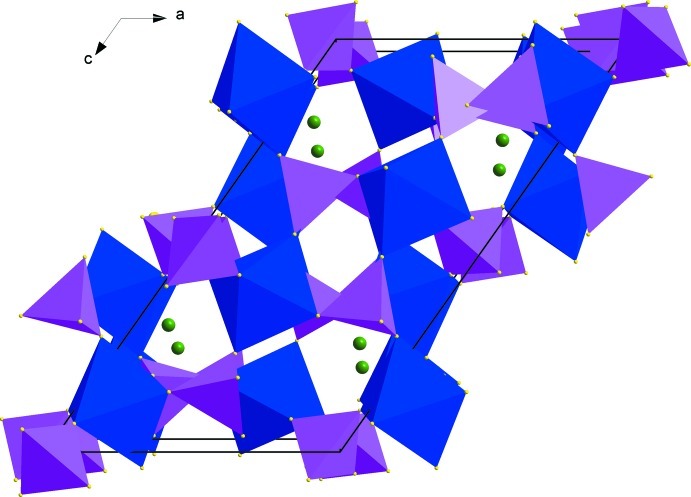
View of Li3V2(PO4)3 down the b axis. VO6 octahedra are shown in blue and PO4 tetrahedra are shown in pink.

## supplementary crystallographic information

### Comment

Trilithium divanadium(III) tris(orthophosphate), Li_3_V_2_(PO_4_)_3_, has been investigated as a cathode material of secondary batteries (Yin *et al.*, 2003) and its structure has been reported based on powder diffraction data (Yin *et al.*, 2003; Patoux *et al.*, 2003; Kuo *et al.*, 2008; Yang *et al.*, 2010; Fu *et al.*, 2010). In an attempt to prepare new mixed-metal phosphates using LiCl as a flux, we were able to isolate crystals of Li_3_V_2_(PO_4_)_3_, and report here the results of the structure analysis based on single-crystal X-ray diffraction data.

The title compound adopts the Li_3_Fe_2_(PO_4_)_3_ structure type. The general structural features of this compound are the same as reported previously (Patoux *et al.*, 2003; Fu *et al.*, 2010). However, as would be expected, the bond lengths found here from single-crystal diffraction data are more accurate than those reported previously from powder diffraction data. For example, the V—O distances (Table 1) reported by Kuo *et al.* (2008) range from 1.846 (3) to 2.258 (4) Å compared with 1.904 (2)–2.117 (2) Å here. Figure 1 shows the local coordination environment of the V and P atoms. In the structure, VO_6_ octahedra are joined to PO_4_ tetrahedra forming a [V_2_(PO_4_)_3_] unit. These units share a terminal oxygen atom to construct the anionic three-dimensional framework, _∞_^3^[V_2_(PO_4_)_3_]^3-^ (Fig. 2). The V—O distances are in good agreement with those calculated from their ionic radii (1.99 Å, Shannon, 1976), assuming a valence of +III for V.

The Li^+^ ions in the empty channels are surrounded by four O atoms in distorted tetrahedral coordination sites. There are three crystallographically independent Li sites for this phase. It has been reported that the positions of the Li atoms can vary depending on the synthetic conditions while those of the V, P, and O atoms comprising the rigid framework remain intact (Yang *et al.*, 2010). The Li positions found from the present single-crystal study are consistent with those reported by Patoux *et al.*(2003) and of a sample treated with microwave radiation at 1123 K for 3 min by Yang *et al.* (2010). According to bond valence sum calculations (Adams, 2001) for the various structure determinations, our study gives the lowest global instability index, *Gii* = 0.027. The *Gii* values of structures with Li positions similar to ours are likewise relatively low (*i.e.* 0.079; Yang *et al.*, 2010), while those with Li positions considerably different as those from the present structures are much higher (*i.e.* 0.175; Yin *et al.*, 2003).

The classical charge balance of the title compound can be represented as [Li^+^]_3_[V^3+^]_2_[P^5+^]_3_[O^2-^]_12_.

### Experimental

The title compound, Li_3_V_2_(PO_4_)_3_, was prepared by the reaction of the elements with the use of the reactive halide-flux technique. A combination of the pure elements, Nb powder (Alfa Aesar 99.8%), V powder (STREM CHEMICALS 99.5%) and P powder (CERAC 99.5%) were mixed in a fused silica tube in a molar ratio of Nb:V:P = 1:1:3 and then LiCl (Sigma-Aldrich 99%) was added. The mass ratio of the reactants and the halide was 1:5. The tube was evacuated to 0.133 Pa, sealed, and heated gradually (150 K/h) to 1123 K, where it was kept for 12 h. The tube was cooled to room temperature at a rate of 3 K/h. The excess halide was removed with water and colourless block-shaped crystals were obtained. The crystals are stable in air and water. A qualitative X-ray fluorescence analysis of selected crystal indicated the presence of V, P, and O. The composition of the compound was determined by single-crystal X-ray diffraction.

### Refinement

Although all the previous structural studies of Li_3_V_2_(PO_4_)_3_ have been performed in space group settings P<ι>112_1_/n<ι> or P<ι>12_1_/n<ι>1 of space group no. 14, we have chosen the standard setting, P<ι>12_1_/c<ι>1, for this and future studies. For the comparison between the different settings in this and the previous studies, the fractional coordinates transformed to the standard setting for the various entries in the ICSD (2012) can be used. The highest peak (0.58 e/Å-3) and the deepest hole (-0.49 e/ Å-3) are 0.68 Å and 0.77 Å from the atom O12 and P1, respectively.

### Crystal data

**Table:**

Li_3_V_2_(PO_4_)_3_	*F*(000) = 784
*M**_r_* = 407.61	*D*_x_ = 3.03 Mg m^−^^3^
Monoclinic, *P*2_1_/*c*	Mo *K*α radiation, λ = 0.71073 Å
*a* = 8.6201 (4) Å	Cell parameters from 5732 reflections
*b* = 8.6013 (4) Å	θ = 3.4–27.6°
*c* = 14.7465 (7) Å	µ = 2.70 mm^−^^1^
β = 125.204 (3)°	*T* = 290 K
*V* = 893.39 (7) Å^3^	Block, colourless
*Z* = 4	0.08 × 0.04 × 0.04 mm

### Data collection

**Table:**

Rigaku R-AXIS RAPID diffractometer	1772 reflections with *I* > 2σ(*I*)
Graphite monochromator	*R*_int_ = 0.031
ω scans	θ_max_ = 27.5°, θ_min_ = 3.4°
Absorption correction: multi-scan (*ABSCOR*; Higashi, 1995)	*h* = −11→10
*T*_min_ = 0.741, *T*_max_ = 1.000	*k* = −10→11
8328 measured reflections	*l* = −19→19
2031 independent reflections	

### Refinement

**Table:**

Refinement on *F*^2^	0 restraints
Least-squares matrix: full	Primary atom site location: structure-invariant direct methods
*R*[*F*^2^ > 2σ(*F*^2^)] = 0.024	Secondary atom site location: difference Fourier map
*wR*(*F*^2^) = 0.062	*w* = 1/[σ^2^(*F*_o_^2^) + (0.0223*P*)^2^ + 1.8904*P*] where *P* = (*F*_o_^2^ + 2*F*_c_^2^)/3
*S* = 1.08	(Δ/σ)_max_ = 0.001
2031 reflections	Δρ_max_ = 0.58 e Å^−^^3^
181 parameters	Δρ_min_ = −0.49 e Å^−^^3^

### Special details

**Table:**

Geometry. All e.s.d.'s (except the e.s.d. in the dihedral angle between two l.s. planes) are estimated using the full covariance matrix. The cell e.s.d.'s are taken into account individually in the estimation of e.s.d.'s in distances, angles and torsion angles; correlations between e.s.d.'s in cell parameters are only used when they are defined by crystal symmetry. An approximate (isotropic) treatment of cell e.s.d.'s is used for estimating e.s.d.'s involving l.s. planes.
Refinement. Refinement of *F*^2^ against ALL reflections. The weighted *R*-factor *wR* and goodness of fit *S* are based on *F*^2^, conventional *R*-factors *R* are based on *F*, with *F* set to zero for negative *F*^2^. The threshold expression of *F*^2^ > σ(*F*^2^) is used only for calculating *R*-factors(gt) *etc*. and is not relevant to the choice of reflections for refinement. *R*-factors based on *F*^2^ are statistically about twice as large as those based on *F*, and *R*- factors based on ALL data will be even larger.

### Fractional atomic coordinates and isotropic or equivalent isotropic displacement parameters (Å^2^)

**Table:**

	*x*	*y*	*z*	*U*_iso_*/*U*_eq_	
Li1	0.1133 (8)	0.5883 (7)	0.1934 (4)	0.0266 (12)	
Li2	0.1891 (9)	0.1919 (7)	0.2599 (5)	0.0356 (15)	
Li3	0.4730 (7)	0.2213 (6)	0.1767 (4)	0.0186 (10)	
V1	0.13814 (6)	0.52846 (5)	0.38977 (4)	0.00672 (11)	
V2	0.36217 (6)	0.53898 (5)	0.11037 (3)	0.00643 (11)	
P1	0.04417 (9)	0.25109 (7)	0.00782 (5)	0.00655 (14)	
P2	0.45782 (9)	0.39759 (8)	0.35181 (5)	0.00672 (14)	
P3	0.75192 (9)	0.38467 (7)	0.14738 (5)	0.00638 (14)	
O1	0.0267 (3)	0.1788 (2)	0.09643 (16)	0.0126 (4)	
O2	0.0361 (3)	0.3649 (2)	0.42742 (16)	0.0150 (4)	
O3	0.0850 (2)	0.0021 (2)	0.28038 (15)	0.0102 (4)	
O4	0.1152 (3)	0.6330 (2)	0.06577 (16)	0.0111 (4)	
O5	0.1785 (3)	0.7151 (2)	0.31962 (15)	0.0109 (4)	
O6	0.2392 (2)	0.3319 (2)	0.07040 (16)	0.0112 (4)	
O7	0.2789 (3)	0.3861 (2)	0.35185 (16)	0.0125 (4)	
O8	0.3675 (3)	0.5514 (2)	0.54043 (16)	0.0171 (4)	
O9	0.4764 (3)	0.2357 (2)	0.31413 (15)	0.0099 (4)	
O10	0.5906 (3)	0.0200 (2)	0.23807 (15)	0.0116 (4)	
O11	0.5994 (3)	0.4098 (2)	0.16881 (15)	0.0110 (4)	
O12	0.6748 (3)	0.4125 (2)	0.02723 (15)	0.0128 (4)	

### Atomic displacement parameters (Å^2^)

**Table:**

	*U*^11^	*U*^22^	*U*^33^	*U*^12^	*U*^13^	*U*^23^
Li1	0.030 (3)	0.038 (3)	0.017 (3)	−0.008 (3)	0.017 (2)	−0.005 (2)
Li2	0.042 (3)	0.030 (3)	0.022 (3)	−0.020 (3)	0.010 (3)	−0.001 (2)
Li3	0.018 (2)	0.016 (2)	0.021 (3)	0.003 (2)	0.011 (2)	0.004 (2)
V1	0.0067 (2)	0.0061 (2)	0.0080 (2)	−0.00005 (16)	0.00464 (17)	0.00055 (15)
V2	0.0069 (2)	0.0060 (2)	0.0073 (2)	−0.00006 (15)	0.00458 (17)	0.00017 (15)
P1	0.0062 (3)	0.0055 (3)	0.0086 (3)	0.0001 (2)	0.0046 (2)	0.0000 (2)
P2	0.0066 (3)	0.0062 (3)	0.0073 (3)	0.0005 (2)	0.0040 (2)	−0.0001 (2)
P3	0.0063 (3)	0.0058 (3)	0.0078 (3)	−0.0002 (2)	0.0046 (2)	−0.0005 (2)
O1	0.0118 (9)	0.0150 (9)	0.0122 (9)	−0.0020 (7)	0.0076 (8)	0.0018 (8)
O2	0.0147 (9)	0.0146 (10)	0.0143 (10)	−0.0023 (8)	0.0076 (8)	0.0053 (8)
O3	0.0106 (8)	0.0081 (8)	0.0093 (9)	0.0027 (7)	0.0042 (7)	0.0009 (7)
O4	0.0104 (8)	0.0107 (9)	0.0133 (9)	0.0034 (7)	0.0074 (8)	0.0015 (7)
O5	0.0163 (9)	0.0075 (9)	0.0119 (9)	−0.0022 (7)	0.0099 (8)	−0.0003 (7)
O6	0.0081 (8)	0.0088 (9)	0.0140 (10)	−0.0015 (7)	0.0048 (8)	−0.0002 (7)
O7	0.0128 (9)	0.0105 (9)	0.0191 (10)	−0.0010 (7)	0.0120 (8)	−0.0031 (8)
O8	0.0094 (9)	0.0205 (10)	0.0131 (10)	−0.0009 (8)	0.0016 (8)	−0.0051 (8)
O9	0.0133 (9)	0.0062 (8)	0.0106 (9)	0.0022 (7)	0.0071 (7)	0.0002 (7)
O10	0.0173 (9)	0.0088 (9)	0.0110 (9)	−0.0016 (7)	0.0096 (8)	−0.0016 (7)
O11	0.0109 (8)	0.0124 (9)	0.0119 (9)	0.0027 (7)	0.0078 (8)	0.0020 (7)
O12	0.0136 (9)	0.0155 (9)	0.0100 (9)	0.0013 (8)	0.0072 (8)	0.0014 (7)

### Geometric parameters (Å, º)

**Table:**

Li1—O4	1.930 (6)	V2—O12^iv^	1.9099 (19)
Li1—O5	1.940 (6)	V2—O6	1.9810 (18)
Li1—O3^i^	2.094 (6)	V2—O4	2.0012 (18)
Li1—O10^ii^	2.215 (6)	V2—O11	2.0316 (18)
Li1—O7	2.584 (6)	V2—O10^ii^	2.0343 (19)
Li2—O3	1.968 (6)	V2—O9^ii^	2.0618 (18)
Li2—O1	1.974 (6)	V2—Li3^ii^	3.032 (5)
Li2—O7	2.004 (6)	P1—O2^v^	1.5199 (19)
Li2—O9	2.153 (7)	P1—O4^vi^	1.5297 (19)
Li2—O5^iii^	2.678 (7)	P1—O1	1.5310 (19)
Li3—O6	1.944 (5)	P1—O6	1.5400 (18)
Li3—O10	1.946 (5)	P2—O8^vii^	1.492 (2)
Li3—O11	1.994 (5)	P2—O9	1.5419 (19)
Li3—O9	2.014 (6)	P2—O7	1.5458 (18)
V1—O2	1.9040 (19)	P2—O10^ii^	1.5497 (19)
V1—O8	1.954 (2)	P3—O12	1.5101 (19)
V1—O1^i^	2.0163 (19)	P3—O11	1.5343 (19)
V1—O7	2.0168 (18)	P3—O5^viii^	1.5454 (19)
V1—O5	2.0450 (18)	P3—O3^ii^	1.5506 (18)
V1—O3^i^	2.1172 (18)		
			
O4—Li1—O5	131.6 (3)	O2^v^—P1—O1	114.61 (12)
O4—Li1—O3^i^	135.8 (3)	O4^vi^—P1—O1	112.35 (11)
O5—Li1—O3^i^	80.6 (2)	O2^v^—P1—O6	107.88 (11)
O4—Li1—O10^ii^	80.9 (2)	O4^vi^—P1—O6	110.77 (10)
O5—Li1—O10^ii^	95.2 (2)	O1—P1—O6	106.40 (11)
O3^i^—Li1—O10^ii^	132.1 (3)	O8^vii^—P2—O9	113.49 (11)
O4—Li1—O7	134.8 (3)	O8^vii^—P2—O7	114.38 (12)
O5—Li1—O7	78.88 (19)	O9—P2—O7	104.68 (10)
O3^i^—Li1—O7	71.26 (18)	O8^vii^—P2—O10^ii^	108.68 (12)
O10^ii^—Li1—O7	61.21 (16)	O9—P2—O10^ii^	109.75 (10)
O3—Li2—O1	94.3 (3)	O7—P2—O10^ii^	105.50 (11)
O3—Li2—O7	128.3 (4)	O12—P3—O11	111.68 (11)
O1—Li2—O7	126.8 (3)	O12—P3—O5^viii^	110.34 (11)
O3—Li2—O9	128.1 (3)	O11—P3—O5^viii^	106.92 (10)
O1—Li2—O9	108.5 (3)	O12—P3—O3^ii^	108.32 (11)
O7—Li2—O9	71.9 (2)	O11—P3—O3^ii^	108.17 (11)
O3—Li2—P2	146.7 (3)	O5^viii^—P3—O3^ii^	111.42 (10)
O1—Li2—P2	117.9 (3)	P1—O1—Li2	132.1 (2)
O7—Li2—P2	36.57 (11)	P1—O1—V1^iii^	140.21 (12)
O9—Li2—P2	36.48 (10)	Li2—O1—V1^iii^	87.60 (18)
O3—Li2—O5^iii^	66.43 (19)	P1^ix^—O2—V1	153.03 (13)
O1—Li2—O5^iii^	69.03 (19)	P3^viii^—O3—Li2	109.4 (2)
O7—Li2—O5^iii^	97.6 (3)	P3^viii^—O3—Li1^iii^	128.10 (19)
O9—Li2—O5^iii^	165.3 (3)	Li2—O3—Li1^iii^	103.1 (3)
P2—Li2—O5^iii^	130.8 (3)	P3^viii^—O3—V1^iii^	136.75 (11)
O6—Li3—O10	146.1 (3)	Li2—O3—V1^iii^	84.99 (19)
O6—Li3—O11	84.4 (2)	Li1^iii^—O3—V1^iii^	84.27 (16)
O10—Li3—O11	126.5 (3)	P1^vi^—O4—Li1	108.2 (2)
O6—Li3—O9	100.9 (2)	P1^vi^—O4—V2	147.84 (12)
O10—Li3—O9	83.4 (2)	Li1—O4—V2	101.8 (2)
O11—Li3—O9	108.5 (3)	P3^ii^—O5—Li1	132.7 (2)
O2—V1—O8	94.48 (9)	P3^ii^—O5—V1	136.88 (11)
O2—V1—O1^i^	88.48 (8)	Li1—O5—V1	90.28 (19)
O8—V1—O1^i^	97.50 (8)	P3^ii^—O5—Li2^i^	110.77 (16)
O2—V1—O7	94.88 (8)	Li1—O5—Li2^i^	85.6 (2)
O8—V1—O7	90.02 (8)	V1—O5—Li2^i^	70.11 (15)
O1^i^—V1—O7	171.50 (8)	P1—O6—Li3	121.90 (18)
O2—V1—O5	165.80 (8)	P1—O6—V2	142.76 (11)
O8—V1—O5	98.04 (8)	Li3—O6—V2	94.12 (17)
O1^i^—V1—O5	83.28 (8)	P2—O7—Li2	92.9 (2)
O7—V1—O5	91.80 (8)	P2—O7—V1	136.13 (12)
O2—V1—O3^i^	90.55 (8)	Li2—O7—V1	129.4 (2)
O8—V1—O3^i^	172.14 (8)	P2—O7—Li1	89.28 (15)
O1^i^—V1—O3^i^	88.65 (8)	Li2—O7—Li1	98.8 (2)
O7—V1—O3^i^	83.53 (8)	V1—O7—Li1	74.63 (14)
O5—V1—O3^i^	77.75 (7)	P2^vii^—O8—V1	168.23 (14)
O12^iv^—V2—O6	98.48 (8)	P2—O9—Li3	118.30 (18)
O12^iv^—V2—O4	93.92 (8)	P2—O9—V2^viii^	136.43 (11)
O6—V2—O4	88.86 (8)	Li3—O9—V2^viii^	96.15 (16)
O12^iv^—V2—O11	94.63 (8)	P2—O9—Li2	87.4 (2)
O6—V2—O11	82.50 (8)	Li3—O9—Li2	105.6 (2)
O4—V2—O11	168.64 (8)	V2^viii^—O9—Li2	109.31 (19)
O12^iv^—V2—O10^ii^	171.87 (8)	P2^viii^—O10—Li3	113.29 (19)
O6—V2—O10^ii^	89.34 (8)	P2^viii^—O10—V2^viii^	141.17 (12)
O4—V2—O10^ii^	83.96 (8)	Li3—O10—V2^viii^	99.23 (17)
O11—V2—O10^ii^	88.56 (8)	P2^viii^—O10—Li1^viii^	103.99 (17)
O12^iv^—V2—O9^ii^	92.40 (8)	Li3—O10—Li1^viii^	97.5 (2)
O6—V2—O9^ii^	167.76 (8)	V2^viii^—O10—Li1^viii^	91.67 (15)
O4—V2—O9^ii^	96.01 (8)	P3—O11—Li3	117.36 (18)
O11—V2—O9^ii^	91.09 (7)	P3—O11—V2	139.65 (12)
O10^ii^—V2—O9^ii^	80.05 (7)	Li3—O11—V2	91.10 (16)
O2^v^—P1—O4^vi^	104.81 (11)	P3—O12—V2^iv^	166.44 (13)

Symmetry codes: (i) −*x*, *y*+1/2, −*z*+1/2; (ii) −*x*+1, *y*+1/2, −*z*+1/2; (iii) −*x*, *y*−1/2, −*z*+1/2; (iv) −*x*+1, −*y*+1, −*z*; (v) *x*, −*y*+1/2, *z*−1/2; (vi) −*x*, −*y*+1, −*z*; (vii) −*x*+1, −*y*+1, −*z*+1; (viii) −*x*+1, *y*−1/2, −*z*+1/2; (ix) *x*, −*y*+1/2, *z*+1/2.
